# Managing Chronic Low Back Pain with Modified Package of Exercise Therapy Enriched by Psychological Interventions in Computer Based Workers: A New Approach in Developing Countries

**Published:** 2019-05

**Authors:** Lida HOSSEINI, Mahboubeh GHAYOUR NAJAFABADI, Amirhossein MEMARI, Ramin KORDI

**Affiliations:** 1. Nursing Care Research Center (NCRC), School of Nursing and Midwifery, Iran University of Medical Sciences, Tehran, Iran; 2. Department of Physical Education, Faculty of Physical Education and Sport Sciences, University of Tehran, Tehran, Iran; 3. Sports Medicine Research Center, Neuroscience Institute, Tehran University of Medical Sciences, Tehran, Iran

## Dear Editor-in-Chief

Low Back Pain (LBP) is the common chronic pain among computer-based workers that contributes to disability, movement limitation and can lead to severe and long term impairment (1). Prevalence of LBP is high among computer-based workers in developing countries and they face several aspects of stress and pressures (2, 3). For example, more than 60% of office workers in Iran had one episode LBP during their working time and they had 92.1% of LBP during their lifetime (2). Actually, LBP impacts the three core adjustment or outcome domains of pain, physical functioning, and emotional functioning that affect the quality of life. Thus, creating a practical and non-pharmacological solution with low cost is a high priority among computer-based workers.

Recently, researchers have focused on designing exercise training package for the computer-based workers to prevent low back pain without any side-effects. However, there are not so many studies on exercise programs to prevent/treatment of LBP in developing countries among computer-based workers (3). However, some researchers designed and suggested therapeutic packages for this purpose. For example, “intelligent physical exercise training” protocol was designed and used to improve muscle strength and reduce sickness absence and productivity losses. To reach these targets, this protocol must achieve six days a week or a minimum of three hours per week (4). Another protocol was “workplace exercise program” that has an effect on neck and shoulder pain and flexibility in office workers. This protocol was designed based on risks associated with office work including “Chair”, “Monitor and Telephone”, “Keyboard and Mouse” (5). The latest validated package of exercise training was designed for computer-based workers in Malaysia to prevent/treatment of LBP (1). The feasibility of this package was also confirmed in another study (6). The package could reduce the symptoms of pain and discomfort in the trunk muscles, particularly those in the shoulder, neck and lower back regions. It could also reduce the pain experienced during and especially, after working hours. Getting to sleep got more easily and feeling more comfortable during sleep were other positive effects of this package among computer-based workers (1). However, in all of these protocols, they missed the psychosocial aspects of LBP in their package.

LBP is influenced not only by physical parameters, but also by biological, psychological, and social factors (3). Several psychological factors have been linked to the perception and adjustment to chronic pain such as stress, anxiety, depression, and fear avoidance that these lead to avoidance of activities, expectations about increased pain, and more self-reported disability. Since the stress at work has been recognized as a major risk factor for chronic pain in computer-based workers, thus it is important to consider psychological factors as well as physical parameters.

To address the issue, we aimed to extend the package developed by Shariat et al., to a more sophisticated program. Our suggested package includes not only office-based physical exercises but also psycho-social training (stress management training (SMT)) which is designed to improve the flexibility and strength of the trunk muscles and also improve the psycho-social issues for computer-based workers. In this package, SMT program includes knowledge about stress, inner psychological resource (self-efficacy), psychological distress, physiological indicators (such as blood pressure) and job performance. SMT program consists of six sessions and sometimes more than ten and need time at least 50 min for per session but can take 30 min in the workplace. The general plan of each session includes four part: 1: relaxation training (3 min), 2: lecture on a selected topic based on knowledge management stress (15 min) 3: exercise (7 min) and finally question and summary (5 min). Program of managing stress in six sessions includes relaxation technique and basic knowledge about stress, time management, goal setting skill, interpersonal communication skills, causal attribution, irrational-dysfunction belief (Table 1). Professional-efficacy is one aspect of Self-efficacy on the job and accesses using 6-item subscale of the Japanese version of the Maslach burnout inventory-general survey (MBI-GS) (7). In our suggested protocol, exercise will be followed by SMT.

**Table 1:** Stress management training (SMT) program

***Part Session***	***Relaxation training***	***Lecture on knowledge management stress***	***Exercise***	***Question and summary***
Saturday	*	Relaxation technique and basic knowledge	*	*
Monday	*	Time management	*	*
Wednesday	*	Goal setting skill	*	*
Saturday	*	Interpersonal communication skills	*	*
Monday	*	Causal attribution	*	*
Wednesday	*	Irrational-dysfunction belief	*	*
